# Vinpocetine in the treatment of poststroke cognitive dysfunction: A protocol for systematic review and meta-analysis

**DOI:** 10.1097/MD.0000000000013685

**Published:** 2019-02-08

**Authors:** Yang Liu, Yanying Yin, Qiao-li Lu, Ying Dan, Mei-song Xu, Ge Song, Chen Li

**Affiliations:** a Department of Neurology, Fifth Central Hospital of Tianjin, Binhai Hospital of Peking University, Tianjin, 300450; b Department of Clinical Medicine, Xinxiang Medical University, Henan, 453003, China.

**Keywords:** cognitive dysfunction, efficacy, meta-analysis, safety, stroke, systematic review, vinpocetine

## Abstract

**Background:**

Previous clinical trials have reported that vinpocetine can be used for the treatment of cognitive dysfunction. However, its efficacy is still inconclusive. In this systematic review study, we aim to assess its efficacy and safety for the treatment of poststroke cognitive dysfunction (PSCD).

**Methods:**

We will search the following electronic databases from the inception to the present to evaluate the efficacy and safety of vinpocetine for patients with PSCD. These databases include CENTRAL, EMBASE, MEDILINE, CINAHL, AMED, and four Chinese databases. All randomized controlled trials (RCTs) of vinpocetine for PSCD will be considered for inclusion without the language restrictions. The methodological quality of all included RCTs will be evaluated by the Cochrane risk of bias tool. The 95% confidence intervals will be utilized to calculate the continuous data, the mean difference or standard mean difference, and dichotomous data with risk ratio.

**Dissemination and ethics:**

The results of this review will be disseminated through peer-reviewed journals. Its results may provide important evidence for the clinical practice, as well as the future studies. It does not require ethical approval, because this systematic review will not involve the individual data.

**Systematic review registration:**

PROSPERO CRD42018115224.

## 1 Introduction

Stroke is one of the most common conditions among the neurological diseases.^[1–3]^ It is also one of the most common leading causes of death around the world.^[4–6]^ More importantly, those stroke survivors often suffer from lots of neurological deficits, such as limbs paralyze, physical and motor skills impairment, as well as the psychological conditions.^[7–11]^ Of those disorders, cognitive impairment is one of the most common deficits caused by stroke.^[11,12]^ It has been estimated that about 35% to 70% poststroke survivors can have this condition in the acute and chronic stages after stroke onset.^[13–15]^ If this condition can not be treated effectively and adequately, it may lead to dementia.

Despite the high prevalence of poststroke cognitive dysfunction (PSCD) is still very high, its treatment are still suffered from limited efficacy and poorly supported.^[16,17]^ Vinpocetine has been reported to improve the neurotransmitter production release or concentration in the brain. Some experimental studies in animals have found a beneficial effect of vinpocetine on memory and learning deficits induced by scopolamine and hypoxia.^[18,19]^ In the clinical trial, it also has been reported to enhance memory function in young healthy volunteers.^[20]^ Although previous review has explored the efficacy vinpocetine for treatment of cognitive dysfunction, the efficacy is still inconclusive,^[21]^ and it was conducted 15 years ago in 2003. Further related clinical trials have also been performed after that study.^[22]^ Thus, it is very necessary to conduct the updated systematic review and meta-analysis to evaluate the efficacy and safety of vinpocetine for treating PSCD.

## 2 Methods

### 2.1 Objective

This systematic review and meta-analysis aims to evaluate the efficacy and safety of vinpocetine for the treatment of patients with PSCD.

### 2.2 Study registration

This systematic review protocol has been registered on http://www.crd.york.ac.uk/ PROSPERO with CRD42018115224. It is designed according to the Cochrane Handbook for Systematic Reviews of Interventions and the Preferred Reporting Items for Systematic Reviews and Meta-Analysis Protocol (PRISMA-P) statement guidelines.^[23]^

### 2.3 Inclusion criteria for study selection

#### 2.3.1 Type of studies

This review will only include randomized controlled trials (RCTs) of vinpocetine for patients with PSCD without any language restrictions. Any other studies including Non-RCTs, quasi-RCTs, case studies, and experimental studies will be excluded.

#### 2.3.2 Type of participants

Patients with cognitive dysfunction after stroke, regarding males or females, of any age will be included. However, patients with cognitive dysfunction caused by other disorders, but not the stroke will be excluded.

#### 2.3.3 Type of interventions

Intervention of any type of vinpocetine treatment will be included. However, the combination of vinpocetine with other therapies will be excluded. Control intervention will include placebo, no intervention, or other medications, except the vinpocetine will be considered.

#### 2.3.4 Type of outcome measurements

Primary outcome of cognitive function will be measured by the scales of Mini–mental state examination, Montreal Cognitive Assessment, Functional Independence Measure Scale, or any other functional assessment scales. Secondary outcomes consist of global impression, quality of life, safety and adverse events.

### 2.4 Search methods for the identification of studies

#### 2.4.1 Electronic searches

The following databases for the related trials will be searched from inception to the present. It included Cochrane Central Register of Controlled Trials (CENTRAL, present), EMBASE (1980 to present), MEDLINE (1946 to present), the Cumulative Index to Nursing and Allied Health Literature (CINAHL, 1982 to present), the Allied and Complementary Medicine Database (AMED, 1985 to present), and four Chinese database Chinese Biomedical Literature Database (CBM; 1980 to present), China National Knowledge Infrastructure (which includes the database China Academic Journals) (CNKI; 1980 to present), VIP Information (VIP; 1980 to present), and Wanfang Data (WANFANG;1980 to present).

#### 2.4.2 Search for other resources

The website of clinical registrations will also be searched for ongoing and recently completed studies. In addition, Bibliographic references in relevant publications will also be considered to search in order to avoiding missing any other eligible RCTs.

#### 2.4.3 Search strategy

The detailed search strategy for CENTRAL is presented in Table 1. Similar strategies will be made and applied for any other electronic databases.

**Table 1:**
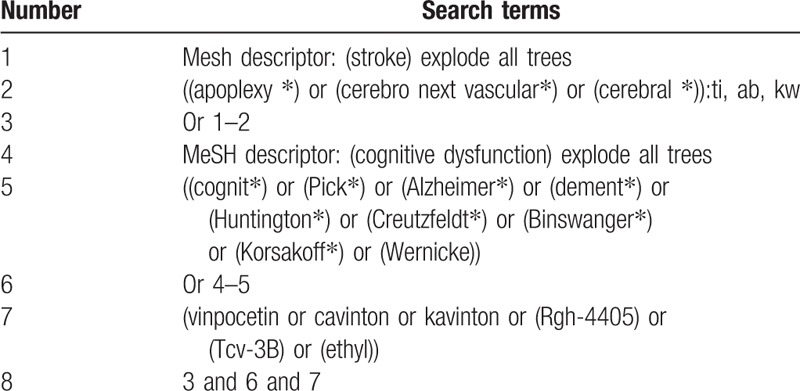
Search strategy utilized in CENTRAL database.

### 2.5 Data collection and analysis

#### 2.5.1 Study selection

Two review researchers will independently review the titles and abstracts of the retrieved potentially eligible studies according to the predefined criteria with a predefined standard form. All the procedure of selections will follow the PRISMA flow chart. If there will be a disagreement between two of them, then a third researcher will be involved to resolve it by through the discussion.

#### 2.5.2 Data extraction and management

Two other review authors will also independently extract data form the included RCTs by using a redefined standard data extraction form, such as author, polished year, country, study design, sample size, and so on. If any disagreement regarding the data extraction will be occurred between two reviewers, a third reviewer will be invited to resolve it by discussion.

#### 2.5.3 Methodological quality assessment of the included studies

Two reviewers will also conduct the quality evaluation by using the Cochrane Handbook for Systematic Reviews of Interventions tool with risk of bias independently. The following 7 domains will be evaluated with the random sequence generation, allocation concealment, blinding of participants/personnel, blinding of outcome assessment, incomplete outcome data, selective outcome reporting, and other bias. Any disagreement will also be solved by a third author involved.

#### 2.5.4 Measurement of treatment effect

As for continuous data, the mean difference (MD) with 95% confidence intervals (CIs) will be utilized. The standardized mean difference (SMD) will be applied if the measurements are not the same tools. As for dichotomous data, the risk ratio (RR) will be performed to conduct the treatment effect with 95% CIs. As for ordinal outcome values, they will also be converted to dichotomous data.

#### 2.5.5 Subgroup analysis

Subgroup analysis will be conducted if the high heterogeneity will be detected, as well as the outcome variable repeatedly. For instance, the different interventions, control treatments, and outcomes.

#### 2.5.6 Dealing with missing data

When the data are insufficient, unclear, or even missing, the original authors will be contact to request those data. If we will not get any reply and additional data can not be achieved, we will analyze the present available data, and the potential impact of those missing data will be discussed as limitations.

#### 2.5.7 Assessment of heterogeneity and data synthesis

The test of *I*^2^ and *χ*^2^ will be applied to quantify inconsistency across studies. If it is possible, meta-analysis will be performed by using random-effect or fixed-effect models. A value of *I*^2^ ≤ 50% (the cut-off point for the present *I*^2^ statistics) will be regarded as having homogeneous. If it is true, a fixed-effect model will be used to pool the data. Otherwise, a random-effect model will be utilized. After that, a narrative summary will be presented if the heterogeneity remains significant.

#### 2.5.8 Publication biases

Funnel plot will be conducted if more than 10 RCTs are available.^[24]^ In addition, Egg's regression will also be used to detect the funnel plot asymmetry.^[25]^

#### 2.5.9 Sensitivity analysis

Where appropriate, sensitivity analysis will be performed to assess the robust of the results based on the methodological qualities, and statistical models.

## 3 Discussion

This study protocol of systematic review and meta-analysis will be conducted to assess the efficacy of vinpocetine treatment to patients with PSCD. Although a previous Cochrane review of vinpocetine for cognitive impairment and dementia has been operated in 2003, the efficacy of vinpocetine is still inconclusive.^[21]^ Additionally, no other review has been updated on this study.^[21]^ Furthermore, no studies specially focused on assessing the efficacy of vinpocetine for patients with PSCD.

Therefore, in the present systematic review and meta-analysis, we will search all related literature without language restrictions to consider including any potential trials of vinpocetine for PSCD. The results of this study will provide a summary of the current evidence on the efficacy and safety of vinpocetine for patients with PSCD. This evidence will also provide useful evidence either for the patients and clinical practice, or the further studies and even the health policy-makers.

## Author contributions

**Conceptualization:** Chen Li, Yang Liu, Ying Dan, Mei-song Xu.

**Data curation:** Chen Li, Yang Liu, Ying Dan, Mei-song Xu, Ge Song.

**Formal analysis:** Yang Liu, Ge Song.

**Investigation:** Ying Dan.

**Methodology:** Yang Liu, Mei-song Xu, Ge Song.

**Project administration:** Chen Li.

**Resources:** Qiao-li Lu, Ying Dan, Mei-song Xu, Ge Song.

**Software:** Yang Liu.

**Supervision:** Qiao-li Lu.

**Validation:** Yanying Yin, Ge Song.

**Visualization:** Yanying Yin, Ge Song.

**Writing – original draft:** Chen Li, Yang Liu, Yanying Yin, Qiao-li Lu, Ying Dan, Mei-song Xu, Ge Song.

**Writing – review & editing:** Chen Li, Yang Liu, Yanying Yin, Qiao-li Lu, Ying Dan, Mei-song Xu, Ge Song.
